# Real‐World Utilisation and Cost Trends of Mineralocorticoid Receptor Antagonists in England: A Population‐Level Prescribing Analysis From 2022 to 2025

**DOI:** 10.1155/cdr/5473277

**Published:** 2026-09-15

**Authors:** Chia Siang Kow, Syed Shahzad Hasan, Kaeshaelya Thiruchelvam, Abdullah Faiz Zaihan

**Affiliations:** ^1^ School of Pharmacy, IMU University, Kuala Lumpur, Malaysia, imu.edu.my; ^2^ School of Applied Sciences, University of Huddersfield, Huddersfield, UK, hud.ac.uk; ^3^ Department of Pharmacy, Shah Alam Hospital, Shah Alam, Selangor, Malaysia

**Keywords:** England, healthcare costs, mineralocorticoid receptor antagonists, prescribing trends, real-world data

## Abstract

**Background:**

Mineralocorticoid receptor antagonists (MRAs) are a cornerstone in the management of cardiovascular and renal diseases. However, the introduction of newer agents and changes in drug pricing may influence prescribing patterns and healthcare expenditure.

**Objective:**

We aimed to evaluate temporal trends in utilisation, cost and cost per item of MRAs in England.

**Methods:**

A retrospective observational study was conducted using publicly available prescribing data from OpenPrescribing, which reports NHS primary care prescribing in England for prescriptions dispensed in the community. Annual data from 2022 to 2025 were analysed for spironolactone, eplerenone and finerenone. Outcomes included total items dispensed, items per 100,000 population, total Actual Cost (£) and cost per item.

**Results:**

Spironolactone remained the most frequently dispensed MRA, with utilisation increasing from 3,140,245 items (5491 per 100,000 population) in 2022 to 4,060,648 (6902 per 100,000) in 2025. Eplerenone utilisation increased from 1,180,762 items (2065 per 100,000) to 1,963,447 (3337 per 100,000). Finerenone demonstrated rapid uptake following minimal initial use, reaching 35,484 items (60.3 per 100,000) in 2025. Total costs for spironolactone increased over the study period, whereas eplerenone costs declined in 2025 despite increased utilisation. Finerenone costs rose in parallel with uptake. Cost per item was lowest for spironolactone from 2022 to 2024, but eplerenone was lower in 2025 (£2.22 vs. £3.13); finerenone remained highest.

**Conclusion:**

Spironolactone remained the most frequently dispensed MRA in England between 2022 and 2025. Eplerenone utilisation increased, whereas its cost per item declined markedly by 2025, and finerenone showed rapid uptake from a very low baseline while retaining the highest cost per item. These descriptive findings identify changing utilisation and expenditure patterns but do not establish indication‐specific use, comparative effectiveness, safety, adherence or cost‐effectiveness and should be considered hypothesis‐generating.

## 1. Introduction

Mineralocorticoid receptor antagonists (MRAs) play a central role in the management of cardiovascular and renal diseases, particularly in patients with heart failure with reduced ejection fraction and chronic kidney disease [1–4]. By inhibiting the effects of aldosterone, MRAs reduce sodium retention, myocardial fibrosis and vascular inflammation, thereby improving clinical outcomes [4–6]. Among available agents, spironolactone has long been established as a cornerstone therapy, supported by robust evidence demonstrating reductions in mortality and hospitalization [1, 2, 7].

Despite its efficacy, spironolactone is associated with endocrine‐related adverse effects due to its non‐selective receptor binding [8, 9]. This has led to the development and use of more selective agents such as eplerenone, which offers improved tolerability, particularly in relation to gynecomastia and sexual dysfunction [9, 10]. More recently, Finerenone has emerged as a novel non‐steroidal MRA with a distinct pharmacological profile and demonstrated cardiorenal benefits in patients with chronic kidney disease and Type 2 diabetes [11–13].

With the expansion of therapeutic options, prescribing patterns for MRAs are expected to evolve over time, influenced by emerging clinical evidence, guideline updates and economic considerations. Although spironolactone remains widely used due to its low cost and established efficacy [14], newer agents such as eplerenone and finerenone may offer advantages in specific patient populations. However, these benefits must be considered alongside their impact on healthcare expenditure, particularly within publicly funded systems such as the National Health Service.

Real‐world data provide valuable insights into how these therapies are adopted in routine clinical practice. In particular, analyses of prescribing trends and associated costs can help to identify shifts in treatment patterns and inform resource allocation. Nevertheless, contemporary data describing the utilisation and economic burden of MRAs at a population level remain limited.

Therefore, this study is aimed at evaluating temporal trends in utilisation, cost and cost per item of spironolactone, eplerenone and finerenone in England using national primary care prescribing data from 2022 to 2025.

## 2. Methods

### 2.1. Study Design and Data Source

This was a retrospective observational study using publicly available prescribing data obtained from OpenPrescribing [15]. OpenPrescribing provides aggregated NHS primary care prescribing data for England, derived from NHS Business Services Authority datasets and covering prescriptions dispensed in the community. The data include the number of prescription items and Actual Cost and can be aggregated at the national level. The dataset does not contain prescribing indications or patient‐level clinical characteristics.

### 2.2. Study Drugs

The analysis included three MRAs: spironolactone (BNF Chemical Code 0202030S0), eplerenone (0202030X0) and finerenone (0202030Y0) [15].

### 2.3. Study Period

Data from January 2022 to December 2025 were included in the analysis. Only complete calendar years were analysed to ensure consistency in temporal comparisons.

### 2.4. Outcome Measures

The primary outcomes were as follows:

1. Total number of items dispensed annually
2. Total annual Actual Cost (£)

Actual Cost, rather than Net Ingredient Cost (NIC), was used for expenditure analyses. Actual Cost is derived from Basic NIC after applicable discount deductions, with specified additions such as payments for consumables, out‐of‐pocket expenses and containers. NHS Business Services Authority changed the discount methodology used in Actual Cost calculations from April 2024 [16]. Secondary outcomes were items dispensed per 100,000 population and cost per item. Population‐standardised utilisation was calculated using Office for National Statistics mid‐year population estimates for England for 2022–2025 [17, 18]. The 2022–2024 denominators used the revised estimates published alongside the mid‐2025 release. Cost per item was calculated as total annual Actual Cost divided by the total number of items dispensed for each medication.

### 2.5. Data Processing and Analysis

Data were extracted and aggregated at the England level by summing values across all commissioning regions for each year. Descriptive analyses were performed to evaluate temporal trends in absolute and population‐standardised utilisation, expenditure and cost per item for each medication. Year‐on‐year percentage changes were calculated where informative. No inferential statistical analyses were conducted, as the study is aimed at describing population‐level prescribing patterns rather than assess causal associations.

## 3. Results

### 3.1. Overall Utilisation Trends

Between 2022 and 2025, absolute and population‐standardised utilisation patterns were assessed for spironolactone, eplerenone and finerenone (Table 1).

**Table 1:** Annual utilisation and cost of mineralocorticoid receptor antagonists in England (2022–2025).

Agent and metric	2022	2023	2024	2025
**Spironolactone—items**	3,140,245	3,407,855	3,745,259	4,060,648
Items per 100,000	5490.93	5876.55	6390.45	6901.78
Actual Cost (£)	6,455,603	11,399,837	12,181,183	12,693,449
Cost per item (£)	2.06	3.35	3.25	3.13
**Eplerenone—items**	1,180,762	1,407,482	1,682,015	1,963,447
Items per 100,000	2064.64	2427.08	2869.98	3337.22
Actual Cost (£)	6,130,257	7,471,175	8,062,028	4,350,782
Cost per item (£)	5.19	5.31	4.79	2.22
**Finerenone—items**	19	1492	12,002	35,484
Items per 100,000	0.03	2.57	20.48	60.31
Actual Cost (£)	1009	47,576	411,085	1,201,326
Cost per item (£)	53.12	31.89	34.25	33.86

*Note:* England population denominators were 57,189,701 (2022), 57,990,791 (2023), 58,607,170 (2024) and 58,834,812 (2025), based on Office for National Statistics mid‐year estimates [17, 18]. The 2022–2024 values incorporate revisions published alongside the mid‐2025 estimates.

Spironolactone remained the most frequently dispensed agent throughout the study period. Total items increased steadily from 3,140,245 in 2022 to 3,407,855 in 2023, 3,745,259 in 2024 and 4,060,648 in 2025. Corresponding population‐standardised utilisation increased from 5491 to 5,877, 6,390, and 6902 items per 100,000 population, respectively.

Eplerenone demonstrated a similar upward trajectory, although at a lower magnitude. Utilisation increased from 1,180,762 items in 2022 to 1,407,482 in 2023, 1,682,015 in 2024 and 1,963,447 in 2025. Corresponding rates were 2065, 2427, 2870 and 3337 items per 100,000 population, respectively.

Finerenone utilisation was negligible in 2022, with only 19 items dispensed (0.03 items per 100,000 population). A marked increase was observed subsequently, rising to 1492 items in 2023 (2.57 per 100,000), 12,002 in 2024 (20.48 per 100,000) and 35,484 in 2025 (60.31 per 100,000), indicating rapid uptake from a very low baseline.

### 3.2. Cost Trends

Total annual Actual Cost for spironolactone increased progressively over time. Costs rose from £6,455,603 in 2022 to £11,399,837 in 2023, followed by £12,181,183 in 2024 and £12,693,449 in 2025. The largest increase occurred between 2022 and 2023: Dispensing volume increased by 8.5%, whereas Actual Cost increased by 76.6% and cost per item by 62.6%.

Eplerenone expenditure increased from £6,130,257 in 2022 to £7,471,175 in 2023 and peaked at £8,062,028 in 2024 before declining substantially to £4,350,782 in 2025 despite continued growth in dispensing volume.

Finerenone expenditure increased markedly in parallel with utilisation. Total Actual Cost rose from £1009 in 2022 to £47,576 in 2023, followed by £411,085 in 2024 and £1,201,326 in 2025.

### 3.3. Cost per Item

Substantial variation in cost per item was observed across the three agents and over time.

For spironolactone, cost per item increased from £2.06 in 2022 to £3.35 in 2023, a 62.6% increase. This was followed by a modest decline to £3.25 in 2024 and £3.13 in 2025, although values remained higher than in 2022.

Eplerenone cost per item was £5.19 in 2022 and £5.31 in 2023, before decreasing to £4.79 in 2024. A pronounced reduction was observed in 2025, when cost per item fell to £2.22, below the corresponding spironolactone value of £3.13.

Finerenone exhibited the highest cost per item across all years. The 2022 value was £53.12, but this estimate was based on only 19 dispensed items and should therefore be interpreted cautiously. Cost per item was £31.89 in 2023, £34.25 in 2024 and £33.86 in 2025.

### 3.4. Comparative Cost per Item

Spironolactone had the lowest observed cost per item from 2022 through 2024. In 2025, eplerenone had a lower cost per item than spironolactone (£2.22 vs. £3.13).

The reversal in 2025 reflected the marked decline in eplerenone cost per item rather than a decline in its dispensing volume.

Finerenone remained substantially more expensive on a per‐item basis than both steroidal MRAs from 2023 onwards, although cost per item should not be interpreted as a standardised treatment cost because the quantity, strength and duration represented by a prescription item may differ between medicines.

### 3.5. Relative Contribution to Utilisation and Expenditure

Spironolactone accounted for the majority of total items dispensed across all years, indicating its dominant share of observed MRA dispensing volume.

Eplerenone contributed a smaller proportion of total utilisation but represented a relatively higher proportion of total expenditure between 2022 and 2024, consistent with its higher observed cost per item during those years. In 2025, its cost per item fell below that of spironolactone.

Finerenone contributed minimally to total utilisation but demonstrated a progressively increasing share of total expenditure over time. Despite its relatively low dispensing volume compared with the other agents, its substantially higher cost per item resulted in a disproportionate contribution to expenditure, particularly in 2024 and 2025.

## 4. Discussion

This study provides a population‐level description of real‐world utilisation and cost patterns of MRAs in England, demonstrating distinct temporal trends across spironolactone, eplerenone and finerenone between 2022 and 2025.

Spironolactone remained the most frequently dispensed MRA throughout the study period. However, the aggregated OpenPrescribing data do not identify the indication for which each prescription item was issued. Spironolactone is used across multiple cardiovascular and noncardiovascular conditions, and the observed dispensing volume therefore cannot be attributed specifically to heart failure. The present analysis supports only the descriptive conclusion that spironolactone had the highest dispensing volume, and from 2022 to 2024, the lowest observed cost per item among the three MRAs. The 2022–2023 increase in spironolactone expenditure was disproportionate to the increase in volume: Items increased by 8.5%, whereas Actual Cost increased by 76.6% and cost per item by 62.6%. Because this increase preceded the April 2024 change in Actual Cost methodology [16], that methodological change cannot explain the 2022–2023 rise; the aggregated chemical‐level data do not allow the contributions of product pricing, strength or formulation mix or other reimbursement factors to be separated.

Eplerenone utilisation also increased steadily, whereas its cost per item fell sharply in 2025 to £2.22, below spironolactone′s £3.13. This finding warrants careful interpretation. Cost per item is based on prescription items rather than standardised doses or treatment duration, and the aggregated data cannot separate the effects of strength or formulation mix, market pricing or reimbursement changes. In addition, NHS Business Services Authority changed the discount methodology used in Actual Cost calculations from April 2024 [16], which may affect comparisons spanning this period. Although previous studies describe a lower incidence of endocrine‐related adverse effects with eplerenone than with spironolactone [9, 10], tolerability, adherence and clinical outcomes were not measured in the present analysis.

Accordingly, the observed reduction in eplerenone cost per item should not be interpreted as evidence that eplerenone should be prioritised over spironolactone or that it is more cost‐effective. Rather, the combination of rising utilisation and changing cost per item is hypothesis‐generating and supports further patient‐level and product‐level analyses incorporating indication, dose, persistence, safety, clinical outcomes and total healthcare costs.

Finerenone exhibited a distinct pattern characterised by rapid uptake from very low utilisation in 2022. Pivotal trials, including FIDELIO‐DKD and FIGARO‐DKD, and the FIDELITY pooled analysis demonstrated cardiorenal benefits in patients with chronic kidney disease and Type 2 diabetes [12, 13, 19]. NICE subsequently recommended finerenone for specified adults with Stage 3 or 4 chronic kidney disease with albuminuria associated with Type 2 diabetes [20]. These developments provide relevant clinical and policy context for contemporary finerenone use, but the present aggregated data cannot determine the indications for prescribing or establish that the observed increase was caused by trial evidence or guideline adoption.

Finerenone remained substantially more expensive per item than the steroidal MRAs. However, acquisition cost alone does not determine overall economic value. Health‐economic evaluations have examined whether the higher drug cost may be offset by avoidance or delay of kidney and cardiovascular events [21]. The present study cannot assess this question because it contains no patient‐level clinical outcomes, hospitalisation data or wider healthcare costs; it therefore should not be interpreted as a comparative cost‐effectiveness analysis.

Taken together, the three agents showed distinct utilisation and cost trajectories, but these patterns should not be interpreted as evidence of treatment substitution, patient stratification or preferred therapeutic sequencing. Determining which MRA offers the greatest value for a particular indication requires indication‐specific comparative effectiveness and economic data that are not available in this dataset.

From a clinical and policy perspective, the sharp 2025 reduction in eplerenone cost per item and the rapid increase in finerenone utilisation are appropriate targets for further investigation rather than direct prescribing recommendations. Acquisition cost per prescription item should be interpreted alongside indication, dose, treatment duration, clinical outcomes and broader healthcare utilisation.

Overall, the observed patterns describe an evolving MRA prescribing and expenditure landscape in England. They generate hypotheses regarding the drivers and consequences of changing MRA use but do not establish comparative clinical benefit, safety, tolerability or preferred treatment selection.

This study has several strengths and limitations. A key strength is the use of national‐level primary care prescribing data for England, providing a broad overview of real‐world utilisation and expenditure over time. Population‐standardised dispensing rates were also calculated to account for changes in population size. However, the analysis was based on aggregated prescribing data without patient‐level information and could not identify prescribing indication, comorbidities, disease severity, dose, treatment duration, adherence, persistence, safety or clinical outcomes. The data cover NHS primary care prescribing in England for prescriptions dispensed in the community and should not be generalised to secondary‐care use or to Scotland, Wales or Northern Ireland. Cost per item is not a standardised treatment‐cost measure because prescription items may contain different quantities, strengths or formulations. In addition, NHS Business Services Authority changed the discount methodology used in Actual Cost calculations from April 2024 [16], which may affect longitudinal cost comparisons. The population‐standardised rates use the revised Office for National Statistics mid‐year estimates for 2022–2024 published alongside the official mid‐2025 estimate [17, 18]. The very small number of finerenone items in 2022 also makes its 2022 cost‐per‐item estimate unstable. Finally, the aggregated design cannot determine the causes of observed temporal changes or assess comparative effectiveness or cost‐effectiveness; these findings should therefore be considered descriptive and hypothesis‐generating.

## 5. Conclusion

In this population‐level analysis of NHS primary care prescribing in England, spironolactone remained the most frequently dispensed MRA between 2022 and 2025, whereas eplerenone utilisation increased and its cost per item declined markedly by 2025. Finerenone showed rapid uptake from a very low baseline but retained the highest cost per item. These findings demonstrate changing utilisation and expenditure patterns across the MRA class. However, because the data are aggregated and are not linked to clinical indications, patient characteristics, treatment duration or outcomes, they should not be interpreted as evidence of comparative effectiveness, safety, preferred treatment selection or cost‐effectiveness. Further patient‐level studies are needed to determine the clinical and economic implications of these trends.

## Author Contributions

C.S.K. and A.F.Z. contributed to the conception and design of the study. S.S.H., K.T. and A.F.Z. contributed to data acquisition, data management and verification. A.F.Z. performed the statistical analysis and drafted the initial manuscript. C.S.K. contributed to study supervision, interpretation of findings and critical revision of the manuscript for important intellectual content. All authors contributed to manuscript revision.

## Funding

No funding was received for this manuscript.

## Disclosure

All authors approved the final version for submission and agreed to be accountable for the integrity and accuracy of the work. All authors meet the ICMJE authorship criteria.

## Conflicts of Interest

The authors declare no conflicts of interest.

## Data Availability

The data that support the findings of this study are openly available in OpenPrescribing at https://openprescribing.net/.
